# Country of birth affects blood pressure in the French hypertensive diabetic population

**DOI:** 10.3389/fphys.2015.00248

**Published:** 2015-09-03

**Authors:** Sola Aoun Bahous, Frédérique Thomas, Bruno Pannier, Nicolas Danchin, Michel E. Safar

**Affiliations:** ^1^Lebanese American University Medical CenterBeirut, Lebanon; ^2^Centre d'Investigations Préventives et Cliniques (IPC)Paris, France; ^3^Institut National de la Santé et de la Recherche Médicale U 970, Hôpital ManhèsFleury-Mérogis, France; ^4^Cardiology Department, Hôpital Européen Georges-Pompidou, Université Paris-DescartesParis, France; ^5^AP–HP, Diagnostic and Therapeutic Center, Paris Descartes UniversityHôtel-Dieu, Paris, France

**Keywords:** country of birth, hypertension, diabetes mellitus, aortic stiffness, wave reflection, ethnicity

## Abstract

In a population of 56,242 individuals living in France, we showed that individuals born in France have significantly different levels of blood pressure (BP) and cardiovascular (CV) risk factors than African and Asian populations born in their own country but living long-term in France (average duration of stay, 5–10 years). The objective of our study was to investigate the impact of country of birth on BP and CV risk factors in a subpopulation of 9245 patients selected solely on the diagnosis of hypertension, either alone or with simultaneous type 2 diabetes. In the subgroup of individuals with hypertension alone, brachial systolic, diastolic, mean and pulse pressure (PP), heart rate (HR), augmentation index and PP amplification were significantly higher in African-born than French- and Asian-born populations. In the subgroup of individuals with both hypertension and diabetes, only augmentation index, PP amplification and brachial and central PP, but not brachial systolic, diastolic, mean BP, and HR, were elevated when the African-born subgroup was compared to the French- and Asian-born populations. Increased body mass index (BMI), waist-hip ratio (WHR), and deprivation scores, but not increased plasma lipids or glycemia, were consistently associated with the African-born population. The combination of diabetes and hypertension in African populations was associated with increased aortic stiffness and PP, together with greater body weight and WHR. In individuals with increased PP and hence systolic hypertension, increased PP requires systolic BP to be reduced whereas notable reductions in diastolic BP may have deleterious consequences.

## Introduction

National health statistics and prospective epidemiological studies, mainly from the United States, have consistently established that hypertensive cardiovascular (CV) disease, stroke, and coronary heart disease are considerably more prevalent in black than in white populations, with a higher prevalence of cardiac and renal failure in the black population (Chrysant et al., 1979; Dunn et al., 1983; Nichols and O'Rourke, 2006; Carson et al., 2011; Chirinos et al., 2011; Frohlich, 2011). In a more recent study in France (Thomas et al., 2012), age, blood pressure (BP), overweight, obesity, and diabetes mellitus were shown to be significant risk factors associated with hypertension in individuals born in Africa, although no striking evidence of CV or renal structure and function impairments was found. In addition, the characteristic CV profile of Asian-born populations was also an important consideration, with lower body weight and HDL-cholesterol, and higher HR and plasma triglyceride levels. It therefore appeared important to determine the impact of country of birth on the CV risk-profile of French-, African- and Asian-born subgroups all residing long-term in France. Thus, the primary objective of the present study was to determine the hemodynamic and metabolic characteristics of three groups of individuals, all living in France on a long-term basis, but born in three different geographical localities: France, Africa, and Asia.

Effective CV prevention requires multiple risk factors to be addressed simultaneously in order to achieve the most significant reduction in morbidity and mortality in a given population (Safar et al., 2013). In particular, both hypertension and diabetes mellitus are major risk factors for CV diseases. Although interesting results were obtained on this subject in a multi-ethnic study of atherosclerosis (Carson et al., 2011), to date, little has been studied in large populations on the possible consequences of combined chronic treatment of hypertension and type 2 diabetes. This is nevertheless an important consideration because approximately 50% of diabetic individuals have hypertension and 20% of hypertensive patients also have diabetes mellitus (Safar et al., 2013). It therefore appeared useful to investigate how country of birth can affect the presence and potential combination of hypertension and type 2 diabetes. This is a particularly important goal of the present study.

Epidemiological studies in hypertension and diabetes mellitus have shown that factors other than high BP play a significant role in CV morbidity and mortality. These include the conventional risk factors of both diseases, and in particular, the role of gender and the degree of obesity (Egan et al., 2010; Regnault et al., 2012). Assessing these factors was therefore another major objective of this report.

Our study involved three different steps: (1) to define the impact of country of birth (France, country of the African continent, country of the Asian continent) on the hemodynamic and metabolic characteristics of individuals all living in France on a long-term basis; (2) to define the impact of country of birth in the same populations according to the presence of two associated CV risk factors, hypertension and type II diabetes; (3) to determine the factors affecting hypertension according to other specific characteristics of the country of birth, and in particular, the degree of obesity.

## Population and methods

The Clinical and preventive investigation Centers (IPC, *Investigations Préventives et Cliniques*) propose a complementary medical examination subsidized by the French national healthcare system (Social Security-CNAMTS), once every 5 years to all working and retired individuals and their families, living in Paris and its suburbs. Approximately 25 000 examinations are conducted annually. This therefore provides a large heterogeneous population of adults of both sexes and enables assessment of three populations, as determined by country of birth: country of the African continent, country of the Asian continent, and France.

Our study sample comprises all individuals (*N* = 56,242) who had a standard health checkup at the IPC Center in Paris between January 2008 and December 2011. Country or continent of birth was ascertained from responses to a self-reported questionnaire, coded by a person with experience in data-collection (Table 1). Of the total population, 57.2% of the men and 51.7% of the women were born in France, 26.9% (men) and 28.0% (women) in African countries (including North Africa), and 5.8% (men) and 7.1% (women) in Asia. Study participants who were born in either Africa or Asia, reported having been resident in France for between 5 and 10 years. The remainder reported countries of birth as other European, American, Pacific, or Caribbean countries, but the number of individuals in each subgroup was insufficient for analysis. Mean age of participants was 43.3 ± 13.8 years (men, 43.6 ± 12.8; women, 43.0 ± 15.0) and was comparable between the three main categories of country of birth (Tables 2–**4**).

**Table 1:** **Prevalence of hypertension (N, %) and diabetes (N, %) in the overall population (men and women) according to country of birth (Asian country, African country, or France)**.

	**No hypertension and no diabetes**	**Hypertension and no diabetes**	**Diabetes and no hypertension**	**Diabetes and hypertension**
Asia (*N* = 3171) Men/Women	2297 (72.4%) 1126/1171	625 (19.7%) 362/263	117 (3.7%) 74/43	132 (4.2%) 78/54
Africa (*N* = 13,485) Men/Women	9183 (68.1%) 4915/4268	3315 (24.6%) 2030/1285	386 (2.9%) 244/142	600 (4.4%) 367/233
France (*N* = 26,925) Men/Women	20,994 (78.0%) 12,044/8950	5305 (19.7%) 3498/1807	219 (0.8%) 143/76	407 (1.8%) 284/123

**Table 2:** **Main characteristics (with standard deviation) of the entire population (***N*** = 56,242 men and women) according to country of birth**.

	**Asia**	**Africa**	**France**
*N*	3171	13,484	26,925
Age (years)	44.27 (12.3)	43.80 (13.5)	42.90 (14.0)
Brachial SBP (mm Hg)	125.8 (0.36)	129.6 (0.2)^***^	126.3 (0.1)
Brachial DBP (mm Hg)	76.6 (0.2)^***^	77.9 (0.1)^***^	75.6 (0.1)
Brachial MBP (mm Hg)	93.0 (0.2)^*^	95.1 (0.1)^***^	92.5 (0.1)
Brachial PP (mm Hg)	49.2 (0.2)^***^	51.7 (0.1)^***^	50.7 (0.1)
Central PP (mm Hg)	35.9 (0.2)^***^	37.9 (0.1)^***^	37.0 (0.1)
Heart rate (bpm)	65.6 (0.2)^***^	65.0 (0.1)^***^	62.4 (0.07)
CAI (%)	125.9 (0.1)^***^	131.4 (0.07)^***^	125.0 (0.05)
PP Amplification (%)	0.726 (0.001)^*^	0.731 (0.0004)^***^	0.728 (0.0003)
BMI (kg/m^2^)	24.8 (0.08)	26.4 (0.04)^***^	24.7 (0.03)
WHR	0.877 (0.001)^***^	0.869 (0.0006)^***^	0.856 (0.0004)
TC (mg/dL)	201.3 (0.7)^**^	195.4 (0.3)^***^	203.6 (0.2)
HDL-C (mg/dL)	49.3 (0.2)^***^	50.5 (0.1)^***^	54.2 (0.08)
TG (mg/dL)	120.1 (1.2)^***^	98.8 (0.6)^*^	97.3 (0.4)
Glycemia (mg/dL)	100.1 (0.3)^***^	98.5 (0.2)^***^	96.6 (0.1)
GAMMA-GT (UI/L)	33.9 (1.0)	34.7 (0.5)	33.6 (0.3)
TT for hypertension (%, N)	5.95 (190)	7.79 (1061)	4.89 (1324)
TT for diabetes (%, N)	4.86 (155)	4.60 (626)	1.02 (277)
TT for cholesterol	7.30 (233)	4.65 (633)	4.87 (1319)
EPICE score	38.7 (0.4)^***^	44.4 (0.2)^***^	23.4 (0.1)

* P < 0.05 vs. France;

** P < 0.01 vs. France;

*** *P < 0.0001 vs. France*.

*SBP, Systolic blood pressure; DBP, Diastolic blood pressure; MBP, Mean blood pressure; Brachial PP, Brachial pulse pressure; Central PP, Central pulse pressure; CAI, Carotid augmentation index; BMI, Body mass index; WHR, Waist-Hip ratio; TC, Total cholesterol; HDL-C, HDL-cholesterol; TG, triglycerides; TT, treatment; EPICE score, Evaluation of Precariousness and Inequalities in Health Examination Centers Score*.

BP was measured three times in the right arm using a semi-automatic electronic device (Omron 705-CP-II, Rosny-sous-Bois, France) with the patient in the supine position after a 10-min period of rest (Nichols and O'Rourke, 2006); the BP value retained was the average of the three measurements. Brachial pulse pressure (PP) [PP = systolic BP (SBP) – diastolic BP (DBP)] and mean BP (MBP = DBP + 1/3 brachial PP) were also calculated. Central (carotid) PP (CPP) was estimated according to gender using the equation validated in a previous personal study (Thomas et al., 2012; Safar et al., 2013). Carotid augmentation index (CAI), the currently used index to describe the relative increase in central BP due to pressure wave reflections (magnitude and timing), was also estimated using the equations proposed by Chirinos et al., to account for the role of ethnic differences in supine BP measurements (Chirinos et al., 2011). Pulse pressure (PP) amplification was calculated as the CPP/BPP ratio. There were no direct measurements of cardiac function.

Waist circumference was measured with a non-elastic tape placed midway between the lower rib cage and the iliac crests on the mid-axillary line, with the patient in the standing position; hip circumference was measured at the maximum protuberance of the buttocks. Waist-hip ratio (WHR) was calculated. Standard biological parameters [automated enzymatic method, HITACHI 917 (Roche, Meylan, France); colorimetric method for albumin dosage and hematology, ABX, Pentra 120 (Meylan, France)] were measured under fasting conditions (Thomas et al., 2012; Safar et al., 2013). High-density lipoprotein (HDL)-cholesterol concentrations were calculated by direct enzymatic assay with cyclodextrin (Roche Diagnostics, Basel, Switzerland). Biological analyses (total cholesterol, HDL-cholesterol, triglycerides, glycemia, and Gamma-GT) and clinical parameters [brachial BP, central BP, heart rate (HR), CAI, PP, body mass index (BMI), and WHR] were assessed on the same day as the checkup. HR was determined with a resting electrocardiogram. Information on country of birth, tobacco consumption, physical activity, personal medical history, social and economic deprivation status, assessed with the EPICES (Evaluation of Precariousness and Inequalities in Health Examination Centers) score (Thomas et al., 2012), and current medications was obtained from the self-reported questionnaire.

The IPC Center was given permission by the French Data Protection Authority (CNIL, *Commission Nationale de l'Informatique et des Libertés*) to conduct these analyses. All participants provided written informed consent at the check-up.

## Statistical analysis

Hypertension was defined as brachial SBP ≥140 mmHg and/or DBP ≥90 mmHg with or without antihypertensive drug therapy. Diabetes was defined as fasting glucose ≥126 mg/dL and/or current specific hypoglycemic treatment.

Multiple variance analysis was used to compare groups adjusted for age and gender. Multiple regression logistic models were used to identify predictive factors of systolic hypertension in each country of birth.

All statistical analyses were performed using the SAS statistical software package (version 8.02; SAS Institute, Cary, NC, USA).

## Results

The prevalence of hypertension and diabetes in both sexes according to country of birth is presented in Table 1. The prevalence of hypertension was higher among study participants born in Africa and in Asia than in those born in France (France vs. Africa, *P* < 0.0001; France vs. Asia, no difference; and Africa vs. Asia, *P* < 0.001). The prevalence of diabetes was significantly higher among participants born in Africa and Asia than in those born in France (France vs. Africa, *P* < 0.0001; France vs. Asia, *P* < 0.0001; Africa vs. Asia, NS). The proportion of participants with both hypertension and diabetes was similar among those who were born in Africa or Asia but considerably lower in those born in France.

Table 2 shows the clinical and biological parameters according to country of birth in the overall study population. Compared to individuals born in France, those who were born in Africa had higher brachial SBP, DBP, MBP, brachial and central PP, HR, CAI and PP amplification, BMI, WHR, plasma glucose, triglycerides, and EPICES scores. In contrast, HDL-cholesterol and total cholesterol were seen to be lower in individuals born in African countries than in those born in France. No differences were observed for Gamma-Gt. Results observed in men and women were very similar (data not shown). Compared to individuals born in France, those who were born in Asia had lower brachial and central PP, PP amplification, plasma cholesterol, and HDL-cholesterol levels. In contrast, individuals born in Asia, were seen to have higher brachial DBP, MBP levels, HR, WHR, triglycerides, glycemia, and EPICES scores. Again, similar findings were observed in both women and men (data not shown). Individuals born in Asian countries had significantly lower brachial SBP, DBP, brachial and central PP levels, PP amplification, CAI, HDL-cholesterol, and BMI than those observed in individuals born in African countries. In contrast, and despite lower BMI levels, individuals born in Asia had higher HR, WHR, total cholesterol, triglycerides, and glycemia compared with those born in Africa. The proportion of study participants who were currently on treatment for hypertension, diabetes, or plasma cholesterol are shown in Table 2; it is noteworthy that the country of birth had no effect on these parameters, as can be seen in Tables 3, 4.

**Table 3:** **Main characteristics (with standard deviation) of individuals with hypertension without diabetes (men and women) according to country of birth**.

	**Asia**	**Africa**	**France**
*N*	625	3315	5305
Age (years)	52.3 (11.5)	51.0 (12.5)	52.7 (13.8)
Brachial SBP (mm Hg)	148.8 (0.59)	151.1 (0.25)^***^	148.3 (0.2)
Brachial DBP (mm Hg)	88.2 (0.4)^*^	89.1 (0.2)^***^	87.0 (0.1)
Brachial MBP (mm Hg)	108.4 (0.4)^*^	109.9 (0.2)^***^	107.5 (0.1)
Brachial PP (mm Hg)	60.6 (0.5)	62.0 (0.2)^**^	61.1 (0.2)
Central PP (mm Hg)	46.3 (0.4)	47.5 (0.2)^***^	46.5 (0.1)
Heart rate (bpm)	68.3 (0.5)^***^	67.1 (0.2)^**^	66.1 (0.1)
CAI (%)	129.5 (0.5)^***^	135.9 (0.2)^***^	131.0 (0.1)
PP Amplification (%)	0.757 (0.003)	0.760 (0.001)^***^	0.754 (0.001)
BMI (kg/m^2^)	26.6 (0.2)	27.9 (0.09)^***^	26.9 (0.07)
WHR	0.91 (0.003)^***^	0.90 (0.001)^*^	0.89 (0.001)
TC (mg/dL)	212.2 (1.6)^***^	207.2 (0.7)^***^	218.6 (0.5)
HDL-C (mg/dL)	48.5 (0.6)^***^	51.2 (0.2)^***^	54.9 (0.20)
TG (mg/dL)	138.0 (2.9)^***^	109.2 (1.3)^***^	116.3 (1.0)
Glycemia (mg/dL)	99.2 (0.4)	97.6 (0.2)^***^	99.5 (0.1)
GAMMA-GT (UI/L)	44.4 (3.4)	42.9 (1.5)^**^	48.9 (1.2)
EPICE score	41.2 (0.9)^***^	46.1 (0.4)^***^	24.1 (0.3)

* P < 0.05 vs. France;

** P < 0.01 vs. France;

*** *P < 0.0001 vs. France*.

*SBP, Systolic blood pressure; DBP, Diastolic blood pressure; MBP, Mean blood pressure; Brachial PP, Brachial pulse pressure; Central PP, Central pulse pressure; CAI, Carotid augmentation index; BMI, Body mass index; WHR, Waist-Hip ratio; TC, Total cholesterol; HDL-C, HDL cholesterol; TG, triglycerides; EPICE score, Evaluation of Precariousness and Inequalities in Health Examination Centers Score*.

**Table 4:** **Main characteristics (with standard deviation) of individuals with hypertension and diabetes (men and women), according to country of birth**.

	**Asia**	**Africa**	**France**
*N*	132	600	407
Age (years)	57.09 (10.0)	59.37 (9.9)	59.73 (10.9)
Brachial SBP (mm Hg)	149.8 (1.52)	152.1 (0.7)	150.6 (0.1)
Brachial DBP (mm Hg)	86.1 (0.9)	86.5 (0.4)	87.7 (0.5)
Brachial MBP (mm Hg)	107.3 (1.0)	108.3 (0.5)	108.7 (0.6)
Brachial PP (mm Hg)	63.7 (1.2)	65.6 (0.6)^**^	62.9 (0.7)
Central PP (mm Hg)	47.4 (1.5)	48.9 (0.5)^*^	46.6 (0.6)
Heart rate (bpm)	72.1 (1.40)	71.4 (0.6)	70.4 (0.7)
CAI (%)	130.0 (1.0)^***^	137.3 (0.4)^***^	134.7 (0.5)
PP Amplification (%)	0.738 (0.005)	0.742 (0.002)^*^	0.732 (0.003)
BMI (kg/m^2^)	27.5 (0.4)^***^	29.9 (0.2)	30.3 (0.25)
WHR	0.97 (0.01)^*^	0.95 (0.002)	0.95 (0.004)
TC (mg/dL)	205.4 (4.0)	196.0 (1.8)^***^	210.4 (2.2)
HDL-C (mg/dL)	45.6 (0.3)^**^	46.7 (0.5)^***^	49.9 (0.6)
TG (mg/dL)	184.3 (8.6)^**^	142.2 (4.0)^*^	155.4 (4.9)
Glycemia (mg/dL)	153.7 (4.9)	153.3 (2.3)	150.8 (2.8)
GAMMA-GT (UI/L)	53.0 (10.1)^*^	54.7 (4.7)^**^	83.1 (5.8)
EPICE score	45.8 (2.0)^***^	50.0 (0.9)^***^	29.8 (1.1)

* P < 0.05 vs. France;

** P < 0.01 vs. France;

*** *P < 0.0001 vs. France*.

*SBP, Systolic blood pressure; DBP, Diastolic blood pressure; MBP, Mean blood pressure; Brachial PP, Brachial Pulse Pressure; Central PP, Central pulse pressure; CAI, Carotid Augmentation index; BMI, Body mass index; WHR, Waist-Hip ratio; TC, Total cholesterol; HDL-C, HDL-cholesterol; TG, triglycerides; EPICE score, Evaluation of Precariousness and Inequalities in Health Examination Centers Score*.

Table 3 shows the characteristics of the study participants with hypertension but without diabetes, according to country of birth. When individuals born in France were compared with those born in Africa, the results did not differ from those of the entire population as shown in Table 2. When individuals born in France and Asia were compared, the differences in brachial and central PP, PP amplification and glycemia levels that were observed in the overall population disappeared among hypertensive subjects.

Table 4 presents the characteristics of the study participants (male and female) with both hypertension and diabetes, according to country of birth. The main finding was that brachial SBP, DBP, MBP, and HR did not differ between participants who were born in Asia, Africa, or France, in contrast with the results shown in Tables 2, 3. Brachial and central PP levels were observed to be slightly higher in individuals born in Africa than in those born in France. With regards to CAI, and to a lesser extent PP amplification, results did not differ from those in Tables 2, 3; this finding contrasted with the results observed for brachial SBP, DBP, MBP, and HR. Because individuals with both hypertension and diabetes tended to be slightly older than those with hypertension alone, one could hypothesize that age or presence of diabetes or both factors might contribute to the particular disorders observed in the African population.

Table 5 presents, for the three populations, the factors associated with SBP variability as a function of country of birth. Independently of age and DBP, only two parameters were shown to be important considerations in each of the three populations: gender and the degree of obesity or BMI. These findings were independent of country of birth but differed in their extent. For example, BMI was associated with 0.63, 0.32, and 0.83% of SBP variability for individuals born in Asia, Africa, and France, respectively.

**Table 5:** **Summary of Multiple Regression Models for study of SBP levels according to country of birth in individuals born in Asia, Africa, and France**.

**Parameter**		**Partial *R*^2^**	**Cumulative *R*^2^**	***P***
**ASIA**				
1^*^	DBP (mm Hg)	0.6270	0.6270	< 0.0001
2	Age (years)	0.0185	0.6455	< 0.0001
3	BMI (kg/m^2^)	0.0063	0.6518	< 0.0001
4	Sex (M/F)	0.0048	0.6566	< 0.0001
5	EPICES index	0.0006	0.6572	0.05
6	WHR	0.0006	0.6578	0.04
**AFRICA**				
1	DBP (mm Hg)	0.6230	0.6230	< 0.0001
2	Age (years)	0.0204	0.6434	< 0.0001
3	Sex (M/F)	0.0096	0.6529	< 0.0001
4	BMI (kg/m^2^)	0.0032	0.6561	< 0.0001
5	Glycemia (mg/dL)	0.0010	0.6571	< 0.0001
6	Smoking	0.0009	0.6580	< 0.0001
7	EPICES index	0.0004	0.6584	0.0004
8	Cholesterol	0.0003	0.6587	0.0004
**FRANCE**				
1	DBP (mm Hg)	0.5708	0.5708	< 0.0001
2	Sex	0.0163	0.5871	< 0.0001
3	BMI (kg/m^2^)	0.0083	0.5954	< 0.0001
4	Age (years)	0.0069	0.6023	< 0.0001
5	CAI	0.0012	0.6035	< 0.0001
6	Glycemia (mg/dL)	0.0010	0.6045	< 0.0001
7	EPICES index	0.0006	0.6052	< 0.0001
8	Smoking	0.0009	0.6060	< 0.0001
9	Triglycerides (mg/dL)	0.0003	0.6063	< 0.0001
10	WHR	0.0002	0.6065	0.0004

*Order is shown from 1 to 10*.

*DBP, diastolic blood pressure; BMI, body mass index; CAI, central augmentation index; WHR, waist-hip ratio; EPICE score, Evaluation of Precariousness and Inequalities in Health Examination Centers Score*.

* *Order in which the parameters were retained in the model*.

## Discussion

This study was conducted to assess the hemodynamic, metabolic and arterial parameters of men and women living in France, but according to their place of birth: Africa, Asia, or France. The results focused on two frequently associated populations: hypertensives and type 2 diabetics. Arterial values were determined from standard hemodynamic and metabolic measurements for both men and women, using classically recognized calculations (Egan et al., 2010; Regnault et al., 2012). Differences among such groups in terms of steady BP, CV-risk profiles and pulsatile arterial hemodynamics (e.g., CAI and CPP/BPP ratio) became very apparent when the country of birth “France” was compared to “Africa,” and “Asia.”

One of the main limitations of our study was that we were unable to specifically analyze race or ethnicity since collecting these data is forbidden in France. Many people born in France are of black African or Asian descent. However, the majority of African-born people living in France come from North African countries and are not black. Furthermore, we did not investigate confounding factors such as diet, which are likely to differ between populations, particularly with regards to salt, alcohol, and vegetable intake. Consequently, based on the information available for this study, any wider generalizations may only be suggested with caution, which explains why this report is principally descriptive with statistical estimates. Thus, from the data presented herein, we were able to demonstrate significant associations between country of birth and hemodynamic or metabolic parameters, but not cause-and-effect relationships. Nevertheless, in a previous report (Safar et al., 2013), we showed that 90% of our study participants had the same country of birth as their parents, suggesting similar genetic features according to country of birth.

Most parameters recorded in African-born participants were significantly higher than in participants born in France; total cholesterol and HDL-cholesterol levels were however lower. The same trend was observed for Asian-born participants, though to a lesser extent and with the notable exception of SBP, brachial and central PP, and PP amplification, which were lower. Comparisons between Asian- and African-born participants showed significantly higher HR, plasma triglycerides, total cholesterol, glycemia, and WHR in Asian-born participants, despite a lower BMI. It is important to note that these results all affected the entire population regardless of gender, the presence of normal or high BP levels, and/or diabetes status.

In the present study, we evaluated the potential modulating effects of two specific CV risk factors: hypertension and type 2 diabetes. We found that, in baseline conditions, steady hemodynamic factors such as brachial SBP, DBP, and MBP as well as HR were significantly higher in African-born individuals than in those born in France or Asia. This observation persisted in individuals with hypertension as the sole risk factor (Table 3) but disappeared when hypertension was associated with diabetes (Table 4). In other words, when hypertension and type 2 diabetes are associated, the difference between “France” and “Africa” does not affect steady hemodynamic parameters but rather pulsatile hemodynamics, as was particularly manifest in the CAI and CPP/BPP ratio calculations (Nichols and O'Rourke, 2006; Regnault et al., 2012; Safar et al., 2013).

To understand the full implication of these results, it is important to bear in mind the specific cumulative metabolic burden of type 2 diabetes, fasting plasma glucose and fasting insulin levels, dyslipidemia and metabolic syndrome. This leads essentially to structural and functional arterial wall damage chiefly via oxidative stress, inflammation and advanced glycation end product (AGE) accumulation (Et-Taouil et al., 2003; Jonk et al., 2007; Levy et al., 2008; Gingras et al., 2009). We and others have already demonstrated that these factors are all related to non-enzymatic protein glycation that forms irreversible, very stiff cross-links in tissue proteins predominating in the large artery wall, causing vascular calcification and altering their structure and mechanical function (Et-Taouil et al., 2003; Jonk et al., 2007; Levy et al., 2008; Gingras et al., 2009). Conversely, insulin promotes capillary recruitment and may be associated with dilatory as well as constrictive responses of resistance arteries via endothelium-NO and endothelin release, respectively (Jonk et al., 2007; Levy et al., 2008). These modifications may well act on small and large arteries via several specific pathways which are not those usually associated with the renin-angiotensin or sympathetic nervous systems (Et-Taouil et al., 2003; Jonk et al., 2007; Levy et al., 2008; Gingras et al., 2009). Taken together, these pathways may well explain the majority of the complex relationships that we observed in our study population, particularly those relating to increased arterial stiffness, which was significantly higher in diabetic than in non-diabetic hypertensive individuals, for the same MBP (Safar et al., 2013).

In the present investigation, it is important to note that factors associated with SBP levels were determined in a multifactorial investigation including successively France, Africa and Asia. In addition to age and DBP, two principal parameters were defined as strong contributors to SBP variability: obesity and gender. These two factors were consistently noted as highly significant among the three categories of our study participants born in France, Africa, or Asia. These results concur with previous findings in the literature indicating that the strongest predictors of BP control are independently: body weight, BP, age, and possibly plasma glucose control, but not dyslipidemia (Lloyd-Jones et al., 2000, 2002; Cushman et al., 2002; Dengo et al., 2010; Zhang et al., 2013). Taken together, these findings suggest that a specific strategy primarily involving body weight reduction should be strongly recommended to improve BP control in hypertensive individuals.

## Conclusion and perspectives

The present report has shown that origin of birth has a notable impact on BP and CV risk profiles among French residents. Individuals born in Africa are considerably more likely than other populations to have a higher brachial BP level. In contrast, individuals born in Asia are more likely to have metabolic disorders with greater glycemia, triglyceride and abdominal fat levels, as determined by a larger waist circumference despite lower BMI.

Country of birth is an appropriate parameter to assess CV profiles, particularly in patients with both hypertension and type 2 diabetes. In 2007, the European Societies of Hypertension and Cardiology showed that arterial stiffness may contribute to such CV profiles. First-line treatment of patients with both hypertension and type 2 diabetes should be based on pharmacological compounds that are known to affect NO dysfunction, angiotensin and endothelin blockade, as well as aortic stiffness and vascular rigidity, as indicated above (Protogerou and Safar, 2007; Protogerou et al., 2007; Sowers, 2013): in individuals with systolic hypertension, because PP is increased, reduction of SBP is useful and necessary while a too large reduction of DBP may have resulting deleterious consequences. Finally, in clinical practice, BP control should systematically include assessment of the degree of obesity mass index. Thus, the findings from this study highlight new perspectives for a more personalized approach to antihypertensive and antidiabetic treatments.

## Summary

### Background

Country of birth is an appropriate parameter by which different populations living in France in the same environment can be studied to identify potential differences between individuals born in France, in Asia or in Africa. Compared with people born in France, the impact of country of birth on BP levels and metabolic factors that influence CV risk is markedly greater in individuals born in Africa and lower or identical in those born in Asia. Findings such as these are observed more frequently in populations with hypertension with or without simultaneous type 2 diabetes as risk factors.

### Results

In the population of study participants with hypertension alone, those who were born in Africa were seen to have significantly higher brachial blood pressure, HR, central and brachial pulse pressure, and augmentation index and amplification levels as compared with individuals born in France and in Asia. In people with hypertension and diabetes mellitus, those born in Africa did not have significantly elevated brachial BP and HR levels but significantly elevated central and brachial pulse pressure, augmentation index, and amplification levels were observed. Regardless of the country of birth, alongside effective BP control, appropriate assessment of body weight should be a mandatory component of the management of all hypertensive patients.

### Conclusion

The role of country of birth is a crucial factor in the choice of antihypertensive therapy and the control of body weight in hypertensive and diabetic populations, particularly when the two CV risk factors occur simultaneously and involve pulsatile arterial hemodynamic alterations. In individuals with systolic hypertension, increased PP requires systolic BP to be reduced whereas notable reductions in diastolic BP may have deleterious consequences.

## Conflict of interest statement

The authors declare that the research was conducted in the absence of any commercial or financial relationships that could be construed as a potential conflict of interest.
